# Compound Heterozygous *CHAT* Gene Mutations of a Large Deletion and a Missense Variant in a Chinese Patient With Severe Congenital Myasthenic Syndrome With Episodic Apnea

**DOI:** 10.3389/fphar.2019.00259

**Published:** 2019-03-12

**Authors:** Zhimei Liu, Li Zhang, Danmin Shen, Changhong Ding, Xinying Yang, Weihua Zhang, Jiuwei Li, Jie Deng, Shuai Gong, Jun Liu, Suyun Qian, Fang Fang

**Affiliations:** ^1^Department of Neurology, Beijing Children’s Hospital, Capital Medical University, National Center for Children’s Health, Beijing, China; ^2^Center for Bioinformatics and Computational Biology, Institute of Biomedical Sciences, School of Life Sciences, East China Normal University, Shanghai, China; ^3^School of Statistics, Faculty of Economics and Management, East China Normal University, Shanghai, China; ^4^Department of Pediatric Intensive Care Unit, Beijing Children’s Hospital, Capital Medical University, National Center for Children’s Health, Beijing, China

**Keywords:** *CHAT*, congenital myasthenic syndromes, episodic apnea, large deletion, severe

## Abstract

Congenital myasthenic syndromes (CMSs) are a group of inherited disorders caused by genetic defects in neuromuscular junctions. Mutations in *CHAT*, encoding choline acetyltransferase, cause congenital myasthenic syndrome with episodic apnea (CMS-EA), a rare autosomal recessive disease characterized by respiratory insufficiency with cyanosis and apnea after infections, fever, vomiting, or excitement. To date, no studies have reported deletions comprised of multiple exons. Here, using next generation sequencing, we identified compound heterozygous mutations, namely a large maternally inherited deletion, including exons 4, 5, and 6, and a paternally inherited missense variant (c.914T>C [p.Ile305Thr]) in *CHAT* in a Chinese patient with a severe phenotype of CMS-EA. Furthermore, the large deletion was also validated by real-time fluorescence quantitative polymerase chain reaction. The patient was a 10-month-old boy, who presented with a weak cry and feeding difficulties soon after birth, ptosis at 4 months old, episodic apnea after fever at 9 months old, and respiratory insufficiency with cyanosis and apnea that required intubation after a respiratory tract infection at 10 months old. Unfortunately, he died in the Pediatric Intensive Care Unit soon after hospitalization. The patient’s elder sister had similar clinical manifestations, and she died prior to the age of 2 months old without a diagnosis. Genotype-phenotype correlation analysis revealed that loss-of-function mutations in exons 4–6 of *CHAT* might cause more severe CMS-EA. To our knowledge, this is the first study to show compound heterozygous *CHAT* mutations consisting of a large deletion and missense mutation in a patient with CMS-EA.

## Introduction

Congenital myasthenic syndromes (CMSs) are a group of inherited disorders caused by genetic defects in neuromuscular junctions. CMSs have been recognized as clinical entities since the 1970s (Conomy et al., 1975; Engel et al., 1977) and are classified into the presynaptic, synaptic, or postsynaptic syndrome type according to the involved mutation sites or genes. With the development of next generation sequencing (NGS) technology, over 30 CMS disease-related genes have now been reported, including *CHAT*, *CHRNE*, *COLQ*, *RAPSN*, and so on; of these, *CHAT* accounts for 4–5% (Abicht et al., 1993–2019; Engel, 2018; Rodriguez et al., 2018).

The *CHAT* gene, located on chromosome 10q11.23, encodes choline acetyltransferase (ChAT), which catalyzes the synthesis of the neurotransmitter acetylcholine from acetyl coenzyme A (AcCoA) and choline. In Ohno et al. (2001), *CHAT* mutations were first reported to cause congenital myasthenic syndrome with episodic apnea (CMS-EA), also named familial infantile myasthenia. Usually, CMS-EA manifests at birth or in early infancy with hypotonia, variable eyelid ptosis, severe bulbar weakness causing dysphagia, and respiratory insufficiency with cyanosis and apnea; the crises recur with infections, fever, excitement, vomiting, or overexertion, and can be prevented or mitigated by anticholinesterase drugs (Ohno et al., 2001). To date, more than 40 *CHAT* mutations have been identified to cause CMS-EA (Human Gene Mutation Database [HGMD^®^] Professional version 2018.1). Although some genetic heterogeneity regarding catalytic activity and phenotypic heterogeneity regarding onset, severity of crises, and prognosis have been described, no genotype-phenotype correlation has been identified. Here, we present the case of a 10-month-old Chinese boy with compound heterozygous *CHAT* variants, including a large deletion (exons 4, 5, and 6) and a missense variant c.914T>C (p.Ile305Thr), which manifested as severe CMS-EA.

## Materials and Methods

### Ethics Statement

The present study was approved by the Ethics Committee of Beijing Children’s Hospital, Capital Medical University, Beijing, China, and was conducted according to the principles expressed in the Declaration of Helsinki. Participants and/or their legal guardians involved in this study gave a written informed consent prior to inclusion in the study. Participants and/or their legal guardians also provided their written informed consent for the material to appear in *Frontiers in Pharmacology* and associated publications without limit on the duration of publication.

### Sample Collection and Library Preparation

The present study included DNA samples from three family members, the parents and the proband. Genomic DNA was isolated using a blood DNA extraction kit according to the manufacturer’s recommendations (Beijing ComWin Biotech Co., Ltd., Beijing, China). A minimum of 3 μg DNA was used to make the indexed Illumina libraries according to the manufacturer’s protocol. The 300–400 bp library size including adapter sequences was finally selected.

### Targeted NGS

Targeted sequencing was performed on the whole mitochondrial genome and 1,033 nuclear genes (Supplementary Table S1), that affect mitochondrial structure and function, or cause some disease difficult to differentiate from mitochondrial disease, such as Krabbe disease, succinic semialdehyde dehydrogenase deficiency, CMS-EA, and so on (Fang et al., 2017).

### Sanger Sequencing

The variant prioritized through NGS was verified by Sanger sequencing in the patient and his parents. The primer sequences used were as follows: F: 5′-GCCGAGAGAAGATCAGCATAAGCA-3′, and R: 5′-GTACAGGTGGAGGTCTCGATCA-3′.

### Reads Mapping and Variant Calling

Paired-end reads of 200 bp (100 bp at each end) from the targeted sequencing were mapped to UCSC human reference genome (GRCh37/hg19) using Burrows–Wheeler Aligner (Li and Durbin, 2010) “mem” mode with default options, followed by removal of polymerase chain reaction (PCR) duplicates and low-quality reads (BaseQ <20). The binary alignment map files were then sorted, indexed, and converted into the mpileup format by SAMtools (Li et al., 2009). Variant calling was implemented in VarScan (Koboldt et al., 2012) software^1^ using the mpileup2snp and mpileup2indel modules.

### Variant Annotation and Prioritization

The identified variants were annotated by ANNOVAR (Wang et al., 2010). The annotation information included minor allele frequency (MAF) in the Genome Aggregation Database (gnomAD) (Lek et al., 2016), variant pathogenicity scores by SIFT (Ng and Henikoff, 2003), PolyPhen2 (Adzhubei et al., 2013), MutationTaster (Schwarz et al., 2010), M-CAP (Jagadeesh et al., 2016), RefSeq gene and the consequences on protein, such as missense, frameshift, in-frameshift, stop-gain, and splicing. Rare variants (MAF < 0.01%) were filtered based on gnomAD (Lek et al., 2016).

### Identification and Quantitative PCR Validation of CHAT Deletion

The CHAT deletion was firstly identified by targeted sequencing data as the loss of heterogeneity in the proband. The read depth for each site (base) within the exons of CHAT gene was calculated. The average read depth of each exon was then calculated by averaging the read depth for the sites within the exon (Figure 3B). Using ALB gene as the internal control, copy numbers of the 4th, 5th, and 6th exons in the *CHAT* gene were estimated by real-time fluorescence quantitative PCR (qPCR) in the patient and his parents.

## Results

### Clinical Features of the Patient

The proband, the second child of two healthy non-consanguineous parents, was a 10-month-old boy. He was born through cesarean section due to a scarred uterus after a full-term pregnancy, with a birth weight of 3.2 kg. Immediately after birth, the boy presented with a weak cry and feeding difficulties, such as slow swallowing, choking easily, and breathing difficulties with apnea if the feeding posture changed, especially when lying down. When he was 4 months old, eyelid ptosis developed. At the age of 9 months, episodic apnea occurred after fever, but improved through symptomatic treatment. The proband’s development milestones were normal.

When the proband was 10 months old, respiratory insufficiency with cyanosis and apnea occurred after a respiratory tract infection, which required mechanical ventilation. A second apneic episode requiring intubation occurred soon thereafter, but the parents refused to permit endotracheal intubation, and thus he received Nasal Continuous Positive Airway Pressure respiratory support. However, repeated episodic apnea continued to occur, and eventually when intubation was deemed necessary. Intravenous immunoglobulin was administered, but was ineffective. The patient’s respiratory function decreased gradually, and he died soon after stopping treatment. Overall, the length of hospital stay was 11 days.

Upon admission to our hospital, the proband’s consciousness was clear. Fluctuating eyelid ptosis was observed, which was aggravated by fatigue. Eye movement in all directions was normal. Limb muscle strength and muscle tone decreased, while the tendon reflex was positive. Meningeal irritation signs and pathological signs were negative.

**FIGURE 1:**
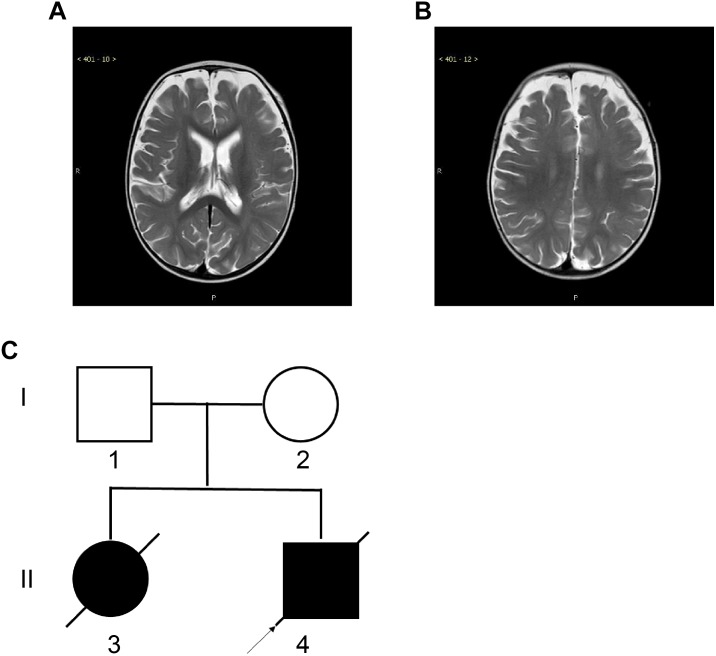
Magnetic Resonance Imaging (MRI) of the proband and the two-generation pedigree. **(A,B)** Brain MRI of the proband at the age of 10 months showed deep sulci in the frontal and parietal lobes and a wide subarachnoid space. **(C)** The two-generation pedigree of the family with CMS-EA. The parents are unaffected, while the two offspring are affected. The arrow indicates the proband.

The neostigmine test was negative, and no anti-acetylcholine receptor or anti-muscle-specific kinase antibodies were detected in the serum. Biochemical examinations, including evaluations of the serum creatine kinase concentration, and serum and cerebrospinal fluid lactate levels, were normal. There were no specific changes in urinary organic acids or blood, as assessed by tandem mass spectrometry analyses. Brain magnetic resonance imaging (MRI) revealed deep sulci in the frontal and parietal lobes and a wide subarachnoid space (Figure 1A,B). Electroencephalography and echocardiography recordings were normal. Pulmonary computed tomography showed slight inflammation, and bronchoscopy did not reveal any abnormalities.

Family history investigation revealed that the proband’s elder sister had similar manifestations and symptoms (Figure 1C), presenting with breathing and feeding difficulties soon after birth. She was hospitalized in the local hospital for nearly 50 days, without a diagnosis, and died of apnea after choking on milk.

### Targeted Sequencing Analysis of the Proband

**FIGURE 2:**
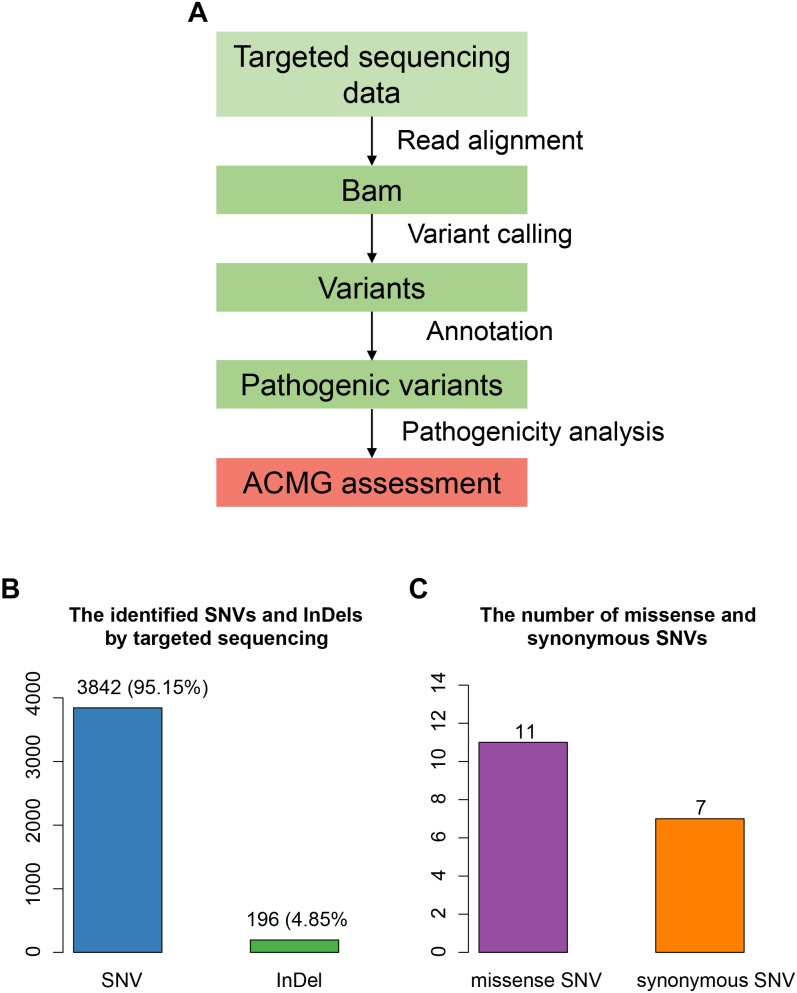
Targeted sequencing-based identification of pathogenic variants. **(A)** Workflow for the analysis of targeted sequencing data. **(B)** The SNVs and InDels identified by targeted sequencing. **(C)** The number of missense and synonymous SNVs in coding regions.

The proband was suspected mitochondrial disease previously, and targeted NGS (Jia and Shi, 2017; Ni and Shi, 2017) was performed on the proband (Figure 2A). In total, we identified 4,038 variants (Figure 2B), including 3,842 single nucleotide variants (SNVs), and 196 small insertions or deletions (InDels). We then annotated these variants using ANNOVAR, and found 979 rare variants (gnomAD MAF < 0.01%). After excluding variants within non-coding regions, we identified only 11 missense variants (Figure 2C).

To evaluate the pathogenicity of these rare missense variants, we performed a systematic pathogenicity analysis using methods described in a previous study (Jin et al., 2018; Yu, 2018). For the missense variants, we identified a missense variant within *CHAT* (c.914T>C [p.Ile305Thr]) as the disease-causing variant (SIFT ≤0.05, PolyPhen ≥0.957, MutationTaster = disease causing, and M-CAP >0.025), which had a homozygous genotype in the proband.

### Validation of the Pathogenic Variants in *CHAT*

**FIGURE 3:**
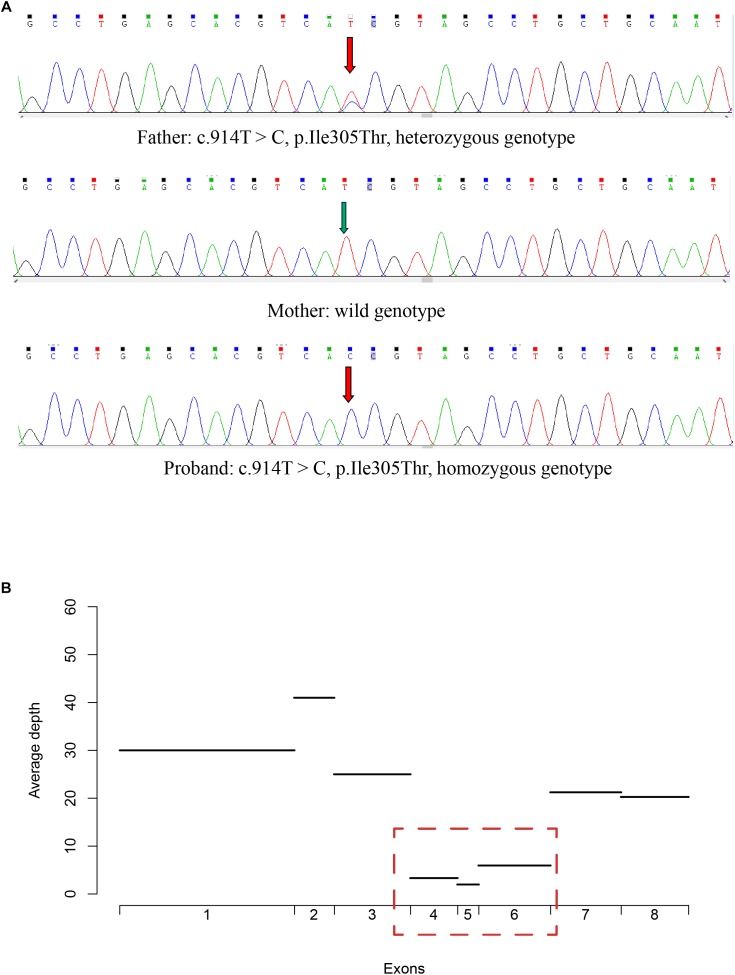
Validation of the missense variant and the large deletion in *CHAT.* **(A)** The genotype of the missense variant in three members of the CMS-EA family, as identified by Sanger sequencing. **(B)** The average read depth for the first 8 exons for the proband was calculated with the targeted sequencing data.

To validate the pathogenic variants, we performed Sanger sequencing on each of the family members. The missense variant in *CHAT* (c.914T>C [p.Ile305Thr]) was validated in the proband, who had a homozygous genotype, and in his father, who had a heterozygous genotype (Figure 3A). However, this variant was absent in the proband’s mother, suggesting that loss of heterozygosity (LOH) may have led to the variant appearing homozygous in the proband.

**FIGURE 4:**
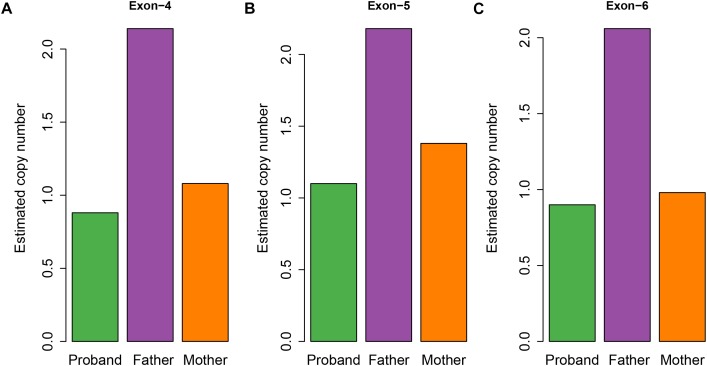
Estimated copy number of the exons for the CMS family members. The estimated copy number of the 4th **(A)**, 5th **(B)**, and 6th **(C)** exons were estimated by qPCR.

To examine whether a large deletion was located within *CHAT*, we calculated the read depth for each exon of *CHAT*. As expectedly, the read depth was significantly reduced from the 4th to 6th exon of the transcript with RefSeq accession number NM_020549 (Figure 3B), indicating that a large deletion was located within these exons. To further validate the large deletion, we performed qPCR to estimate the copy number for exons 4, 5, and 6. In accordance with the read depth analysis, loss of heterozygosity was also observed within regions of the 4th to 6th exon in both the proband and his mother (Figure 4). Hence, the large deletion combined with the missense variant (c.914T>C [p.Ile305Thr]) led to the occurrence of CMS-EA in the proband.

### Functional Characterization of the *CHAT* Variants

**FIGURE 5:**
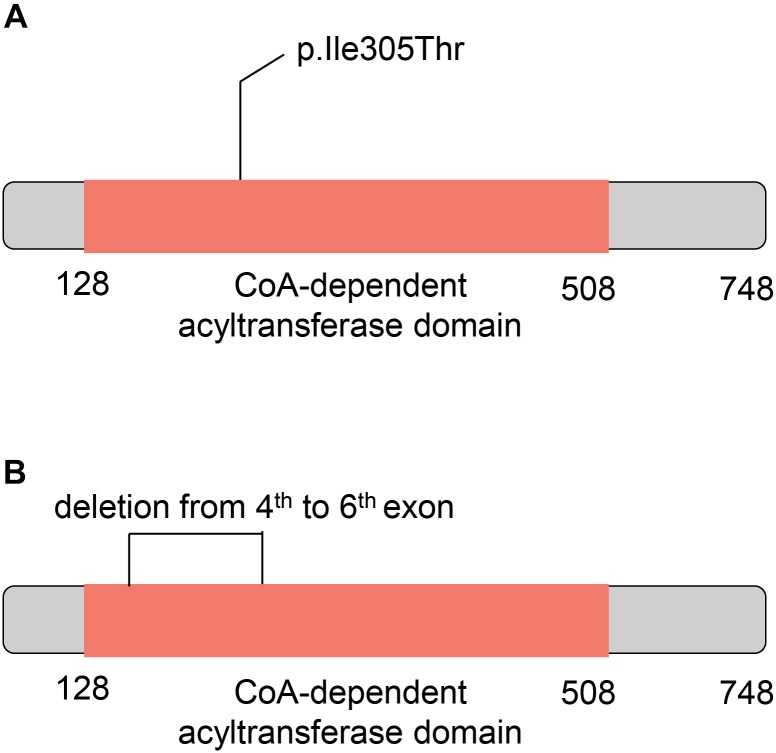
Schematic diagram for the mutant *CHAT* proteins. **(A)** The ChAT protein with c.914T>C (p.Ile305Thr) variant. **(B)** The ChAT protein with the large deletion from the 4th to 6th exons. The red box indicates the acetyl coenzyme -dependent acyltransferase domain.

To further understand the role of the two *CHAT* variants in CMS, we investigated their potential consequences on the ChAT protein. The missense variant in *CHAT* (c.914T>C [p.Ile305Thr]) was located within the CoA-dependent acyltransferase domain (the amino acid sites from 128 to 508) based on the SuperFamily (Pandurangan et al., 2019) annotation (Figure 5A). Moreover, the large deletion located within the 4th to 6th exons also overlapped with the CoA-dependent acyltransferase domain (Figure 5B). As reported in a previous study, the missense mutation markedly reduced ChAT expression in COS cells and had significantly impaired catalytic efficiencies in kinetic studies (Ohno et al., 2001). These results indicated that the CoA-dependent acyltransferase domain might have been disrupted by the two variants in the patient of this study.

## Discussion

Since the first report in Ohno et al. (2001) showing that *CHAT* mutations cause CMS-EA, more than 40 mutations have been found to cause CMS-EA; of these, bi-allelic point mutations are the most frequent (HGMD^®^ Professional version 2018.1). To our knowledge, the present study is the first study to report the case of a patient with CMS-EA caused by compound heterozygous exons deletion and missense mutation in the *CHAT* gene. Our functional characterization results indicated that the CoA-dependent acyltransferase domain might have been disrupted by the two variants in this study. The missense variant has previously been demonstrated to markedly reduce ChAT expression in COS cells and significantly impair catalytic efficiencies in kinetic studies (Ohno et al., 2001). In accordance with this previous report, the severe phenotype of the patient in our study may have been caused by the missense variant combined with the large deletion.

As mentioned earlier, CMS-EA usually presents at birth or in early infancy with hypotonia, ptosis, dysphagia due to severe bulbar weakness, and respiratory insufficiency with cyanosis and apnea. Occasionally, apnea crises may be mistaken for seizures and thus anticonvulsive drugs may be initiated, without positive effects (Schara et al., 2010); as such, EEG is essential for distinguishing apneic crises from seizures, especially video EEG. Several reported patients presented with mental retardation, and MRI showed brain atrophy. There are two possible explanations for this. First, the apnea may have led to brain hypoxemia. Second, mental retardation may be a symptom of ChAT deficiency in the brain (Schara et al., 2010). Indeed, ChAT deficiency has been reported in various developmental and neurodegenerative disorders, including Alzheimer’s disease, Huntington’s disease, amyotrophic lateral sclerosis, Schizophrenia, Rett syndrome, and Sudden Infant Death Syndrome (SIDS) (Oda, 1999). The cause of brain atrophy is undetermined, but normal development milestone in the present study support more that may be induced by brain hypoxemia.

Electrophysiological assessments are important for diagnosing CMS-EA, as repetitive nerve stimulation testing at a low frequency (10 stimuli at 2–3 Hz) might be normal, but prolonged subtetanic stimulation (10 Hz for 5 min) decreased the amplitude of the compound muscle action potential (CMAP) and endplate potential to 50% below baseline (normal decrease is <30%), followed by slow recovery. This slow recovery suggests the presence of a defect in acetylcholine resynthesis and previously aided in the discovery of mutations in *CHAT* (Ohno et al., 2001; Dilena et al., 2014; Engel et al., 2015). Prolonged subtetanic nerve stimulation is rarely used in infants, and thus performing genetic testing as soon as possible is helpful for ensuring early diagnosis and treatment. Because of the severe condition of our patient, electrophysiological assessments were not conducted.

In general, CMS-EA is a treatable rare disease that responds positively to acetylcholinesterase (AchE) inhibitors, and thus early treatment with pyridostigmine is helpful for improving the clinical symptoms and prognosis (Engel, 2018). Although it was reported that midazolam may mitigate the severity of apnea episodes (Mallory et al., 2009; Barisic et al., 2011), further studies are needed. Unfortunately, the symptoms in our patient were so severe that he died before he could receive AchE inhibitor therapy. We think that the findings of this study will help clinicians identify such cases early, perhaps avoiding a negative outcome.

In addition, there are still some limitations in the present study. First, the association between *CHAT* large deletion and poor prognosis requires more cases to support. Moreover, the breakpoint of CHAT deletion was not accurately determined by Sanger sequencing due to lack of more blood sample. To conclude, we first reported compound heterozygous *CHAT* mutations consisting of a large deletion (exons 4, 5, and 6) and missense mutation (c.914T>C [p.Ile305Thr]) in a patient with severe CMS-EA.

## Data Availability

All the clinical data and identified genetic variations have been deposited into the rare disease database, eRAM (Jia et al., 2018), at http://www.pediascape.org/eram/.

## Author Contributions

FF, CD, SQ designed the study. DS, XY, WZ, JwL, JD, SG, and JL collected the data and performed the research, ZL and LZ analyzed the data and wrote the manuscript. All authors reviewed and approved the final manuscript.

## Conflict of Interest Statement

The authors declare that the research was conducted in the absence of any commercial or financial relationships that could be construed as a potential conflict of interest.

## Footnotes

1 http://varscan.sourceforge.net/

## Supplementary Material

TABLE S1 Nuclear gene list of targeted NGS used in the present study.
